# Machine Learning-Assisted DNA Origami Shape Sorting Using Fingerprinting Nanosensors and Feature Engineering

**DOI:** 10.1021/acs.analchem.5c06210

**Published:** 2026-01-12

**Authors:** Shubhajit Singha, M. Mikail Demir, Vinod Morya, Ken Halvorsen, M. Abdullah Canbaz, Arun Richard Chandrasekaran, Mehmet V. Yigit

**Affiliations:** † Department of Chemistry, University at Albany, 1084 State University of New York, Albany, New York 12222, United States; ‡ Department of Information Sciences and Technology, University at Albany, State University of New York, Albany, New York 12222, United States; § The RNA Institute, University at Albany, State University of New York, Albany, New York 12222, United States; ∥ Department of Nanoscale Science and Engineering, University at Albany, State University of New York, Albany, New York 12222, United States

## Abstract

Reconfigurable DNA nanostructures have emerged as a promising research area with applications in drug delivery, molecular computing, biosensing, and stimuli-responsive soft nanomaterials. While significant progress has been made in creating novel DNA nanostructures and exploring their applications, comparatively little effort has focused on developing new methodologies to confirm their folding. Conventional imaging approaches typically rely on sophisticated microscopy techniques including atomic force and transmission electron microscopy. Alternative low-cost methods for verifying DNA nanostructure assembly and shape sorting are thus highly valuable. Here, we present a fingerprinting nanosensor array integrated with machine learning (ML) to distinguish between two DNA origami shapes (triangle and nanotube) and to differentiate them from an unfolded scaffold strand. The nanosensor array, consisting of 11 nanoassemblies, termed nanosensors, is prepared by complexing graphene oxide nanosheets (nGO) with 11 fluorophore-labeled single-stranded DNA probes. Upon complexation, the fluorescence of the DNA probes is quenched through graphene oxide-mediated fluorescence quenching. Adding the DNA nanostructures to each nanosensor displaced a fraction of the surface-adsorbed fluorescent DNA probes, producing unique fluorescence recovery signatures that were subsequently processed through feature engineering for accurate ML-assisted classification. Using this approach, we achieved 94% prediction accuracy in discriminating DNA origami triangle, DNA origami nanotube, and unassembled M13 scaffold. Our strategy provides a new and generalizable platform for shape sorting in DNA origami field, offering new avenues for high-throughput, label-free classification.

## Graphical abstract

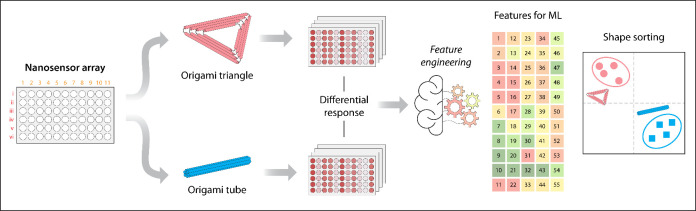

DNA nanotechnology enables the assembly of nucleic acids with nanometer scale accuracy to create sequence-defined shapes.^1,2^ Advances in DNA origami and chemical modifications have enabled the creation of nanometer to micrometer scale structures with different functionalities. As the design strategies and construction capabilities are expanding, there is an increased need for newer techniques to analyze the variety of DNA nanostructures being assembled.^3,4^ Gel electrophoresis remains one of the most common methods,^5^ while more specialized techniques such as atomic force microscopy (AFM) and transmission electron microscopy (TEM) provide high-resolution analysis to visualize and distinguish DNA nanostructures. New methods are being developed for automated, rapid characterization of DNA nanostructures including the use of artificial intelligence.^6^

Machine learning has been used in the optimization of nanoparticle preparation, analysis of nanobio interactions and for applications in nanotheranostics.^7,8^ In DNA nanotechnology, previous works have used convolutional neural network (CNN)^9^ to detect multiple DNA origami structures and estimate their yield using annotated AFM images^10^ and TEM images.^11,12^ Methods based on deep neural networks (DNNs) have also been developed to characterize large fluctuations in dynamic DNA devices.^13^ However, these methods rely on prior characterization and use existing data to process and provide new structural information. A solution-phase approach that can discriminate different DNA nanostructures without requiring specialized and sophisticated techniques remains underexplored.

In this study, we report a nanographene oxide-based sensor array that acts as a chemical nose for the discrimination of DNA origami structures that differ in shape. Recent developments in optical nanosensor arrays have emerged as a powerful and flexible sensing platform, predominantly used for sensing biological markers.^14–18^ The nanosensor arrays reported here are made of two-dimensional graphene oxide nanoparticles (nGO) and fluorescently tagged short single stranded DNAs (fDNA). We have previously used nGO-fDNA assemblies for the detection of biomarkers such as microRNAs, proteins and small molecules.^19–24^

In a conventional nGO-based sensor design, fDNAs are designed to be specific to the target ligand or biomarker. Upon target detection, the surface-adsorbed probe is displaced through target-probe binding, leading to the recovery of fluorescence initially quenched by nGO through graphene oxide-mediated fluorescence quenching.^19–24^ We have reimagined this sensing strategy and developed a new detection mode in which fluorescence is partially recovered when the target molecule displaces a fraction of the surface-adsorbed fDNA in the nGO-fDNA assembly via competitive displacement rather than target-probe binding.^25,26^ Since each target displaces a different fraction of fDNA through this displacement event, the resulting differential response can be utilized for sample discrimination. The fDNAs bind to nGO through a combination of noncovalent interactions, including van der Waals forces, hydrogen bonding, and π–π stacking, that stabilize the nGO-fDNA assembly in aqueous solutions.^26–28^ These fDNAs can detach from the surface when a stronger interaction competitively displaces them either through hybridization with complementary or partially complementary sequences, aptamer–target recognition, or stronger nGO–analyte interactions.^19–24^ Because no specific interaction is present between the fDNAs used in this study and the added DNA origami structures, the displacement observed here arises solely from the DNA origami structures disrupting the nGO-fDNA complex.

To enhance discrimination power, we engineered an array of multiple nanosensors rather than relying on a single nanosensor. Previously, we successfully used this approach for the detection and classification of proteins, bacterial species, and microRNAs, as well as for screening food samples for adulteration in food fraud cases.^26,27,29–31^ In these systems, the nanosensors in the array often generate complex, high-dimensional, and overlapping signals that are difficult to interpret with standard statistical tools.^32^ We used machine learning (ML) to subsequently process this information, where one can spot meaningful trends and relationships even in large, complex, and noisy data sets. By uncovering hidden patterns, reducing noise, and capturing nonlinear behaviors, ML turns raw data into a clear and accurate classification data set for bias-free predictions. However, the predictive power of any ML model is fundamentally dependent on the quality and representation of the input data. To enhance prediction accuracy, feature engineering can be applied to the collected data sets to generate a more diverse set of data points. Feature engineering allows the refinement of raw data sets to introduce more distinct variability and increase the dimensionality that eventually strengthens the ML-model.^33^

Here, we assembled a nanosensor array for discriminating two DNA origami shapes (triangle and nanotube) through fluorescence signal responses that are processed with feature engineering and analyzed by ML for shape sorting (Figure 1). Briefly, nanosensors were assembled using nGO complexed with 11 fDNAs with different sequence information (Table S1). The adsorption of fDNAs on surface quenches the fluorescence signal through graphene oxide-mediated fluorescence quenching (Figure 1a). Our results demonstrate that the DNA origami nanostructure in solution interacts with the nanosensors and displaces a fraction of the surface-adsorbed fDNAs in a manner that correlates with the sequence and length of the fDNAs. This displacement produces a distinct fluorescence recovery profile for each nanosensor in the array (Figure 1b). Each DNA origami structure has a distinct overall dimension, shape and surface features; therefore, the nanosensor array produces a different response for each tested nanostructure. We recorded the fluorescence recovery patterns over a period of 60 min with 2 min time intervals for each origami structure to increase the data set that can be used for discriminating the tested origami structures (Figure 1b). To capture the dynamic evolution of the fluorescence signals, a feature engineering strategy termed the “Slope model” was implemented to discriminate between the different DNA origami shapes through Random Forest (RF) classifier and a Partial Least Squares Discriminant Analysis (PLS-DA) (Figure 1c).

**Figure 1:**
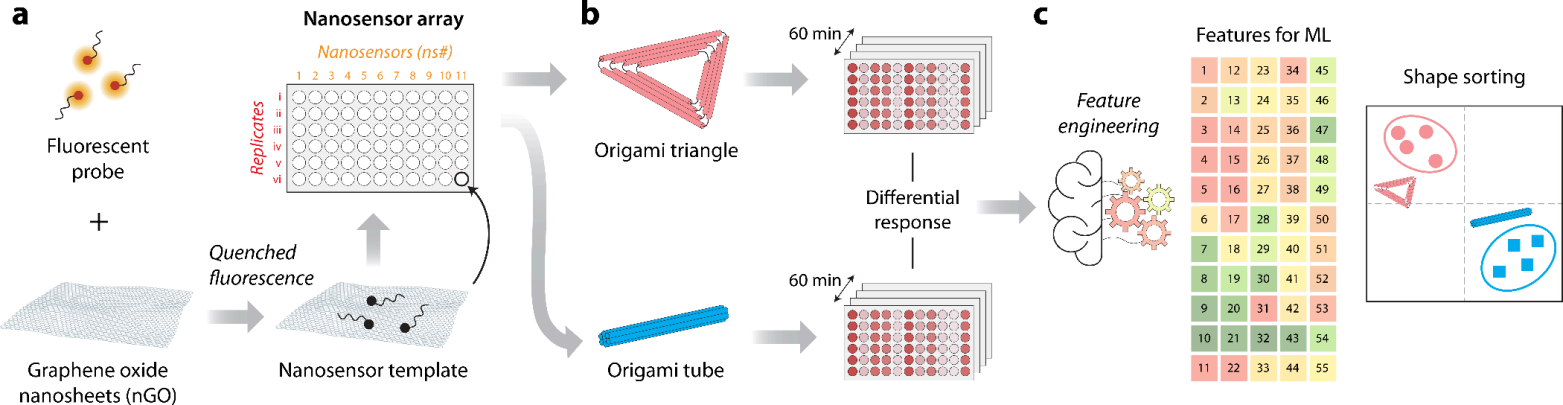
Overview of ML-guided nanosensor array for discriminating two different DNA nanostructures made from the same genetic material. (a) Nanosensor arrays are prepared from a set of fluorophore-tagged DNA probes (fDNAs) and nanographene oxide (nGO). (b) Addition of target DNA origami structures provides a fingerprint fluorescence signal. (c) Machine learning analysis of the fingerprint discriminates DNA nanostructures of different shapes.

In this study, the nGO serves as the template for the nanosensors. Therefore, we first characterized the nGO using UV–visible absorption spectroscopy, which revealed the characteristic absorption peak at ∼230 nm (Figure 2a). Dynamic light scattering measurements indicated an average hydrodynamic diameter of ∼200 nm, consistent with our previous reports (Figure 2b).^19,22–24^ The amount of nGO required for optimal quenching (∼94%) was evaluated across a range of nGO concentrations using 20 nM of a representative fDNA (Figure S1). A concentration of 1.38 μg/mL nGO was found to be sufficient for efficient nanosensor formation. We then validated nGO-based fluorescence quenching for all 11 fDNAs used in this study (representative data in Figure 2c and all sensors in Figure S2). These fDNAs were chosen based on their adsorption and release properties on nGO based on our prior study where we evaluated the interaction of various fDNAs on two-dimensional nanoparticles.^25–27^ After confirming the properties of nGO and successfully demonstrating its quenching properties we then assembled an array of 11 nanosensors by adsorbing the 5′-fluorescein (FAM)-tagged fDNAs on the nGO. We specifically used fDNA strands (polyA, polyC, and polyT) with lengths of 23, 18, 12, and 7 nucleotides. PolyG was intentionally excluded because its strong tendency for tertiary structure formation inhibits effective adsorption onto the nGO surface. Longer DNA sequences exhibit stronger binding to graphene oxide due to a greater number of noncovalent interaction sites, which leads to reduced fluorescence recovery upon target binding. In contrast, shorter sequences bind weakly and therefore display higher fluorescence recovery. Furthermore, the binding affinity of nucleobases to graphene oxide generally follows the order G > A > C ≥ T.^28^ Consequently, the use of different polynucleotides introduces additional variation in binding strength and sensor response. By combining DNA strands with different lengths and base compositions, we generated a nanosensor array capable of producing differential fluorescence responses upon interaction with target biomolecules, thereby enhancing discrimination performance. We optimized the array by ensuring the minimum number of nanosensors required for effective classification and found that an array consisting of 11 nanosensors composed of nGO and different polynucleotides was sufficient to achieve robust discrimination.

**Figure 2:**
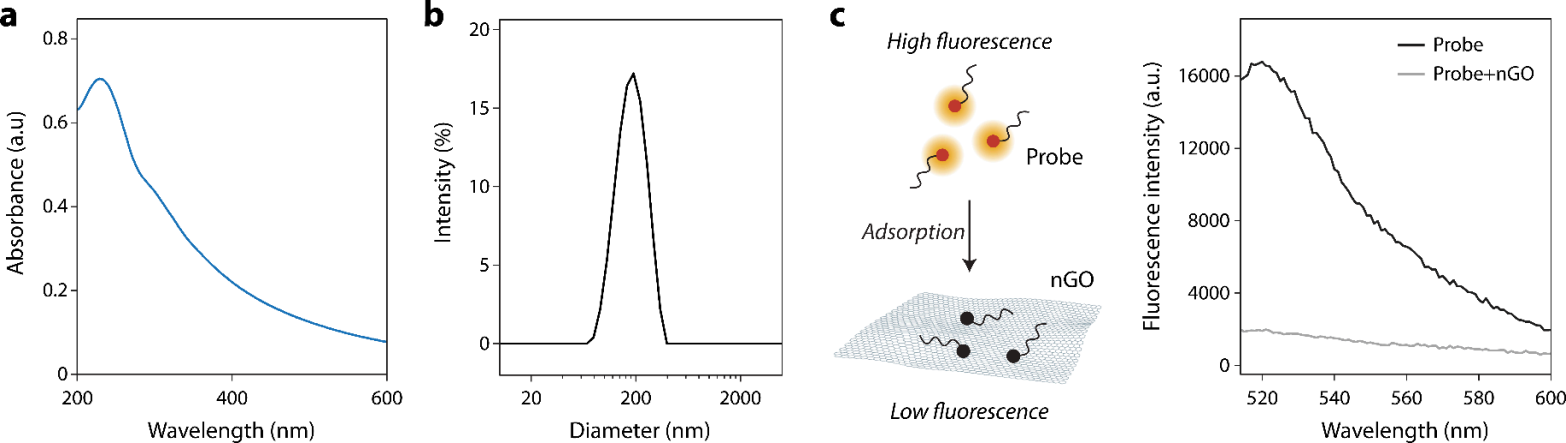
Nanosensor characterization. (a) Absorbance of nGO nanosheets showing characteristic peak at 230 nm. (b) Dynamic light scattering analysis showing the size of nGO particles. (c) Quenching of fluorescence on the addition of a FAM-labeled single stranded DNA probe (fDNA) on nGO sheets. Right: The fluorescence spectra before and after quenching of the fDNA probe.

We then assembled and characterized the DNA origami structures to be discriminated using the nanosensor array. For this study, we selected two model DNA origami nanostructures assembled from the same M13 scaffold: a DNA origami tube (∼400 nm in length) and a DNA origami triangle (∼100 nm per edge).^31,32^ We assembled the DNA origami nanostructures using the M13 scaffold and complementary DNA staple strands (Tables S2–S3), and their proper folding was confirmed by atomic force microscopy (AFM) (Figure 3a). Our lab has extensively studied DNA nanostructures in both fundamental and applied research, allowing us to thoroughly characterize them and interpret the data collected.^34,35^ We chose these structures because they share the same sequence composition, being constructed from an identical scaffold. The DNA origami structures constitute the vast majority of the sample content. Any unincorporated oligonucleotides are present at comparable levels between the two origami constructs. Therefore, we tested our nanosensor array with these minimally perturbed samples without prior purification.

**Figure 3:**
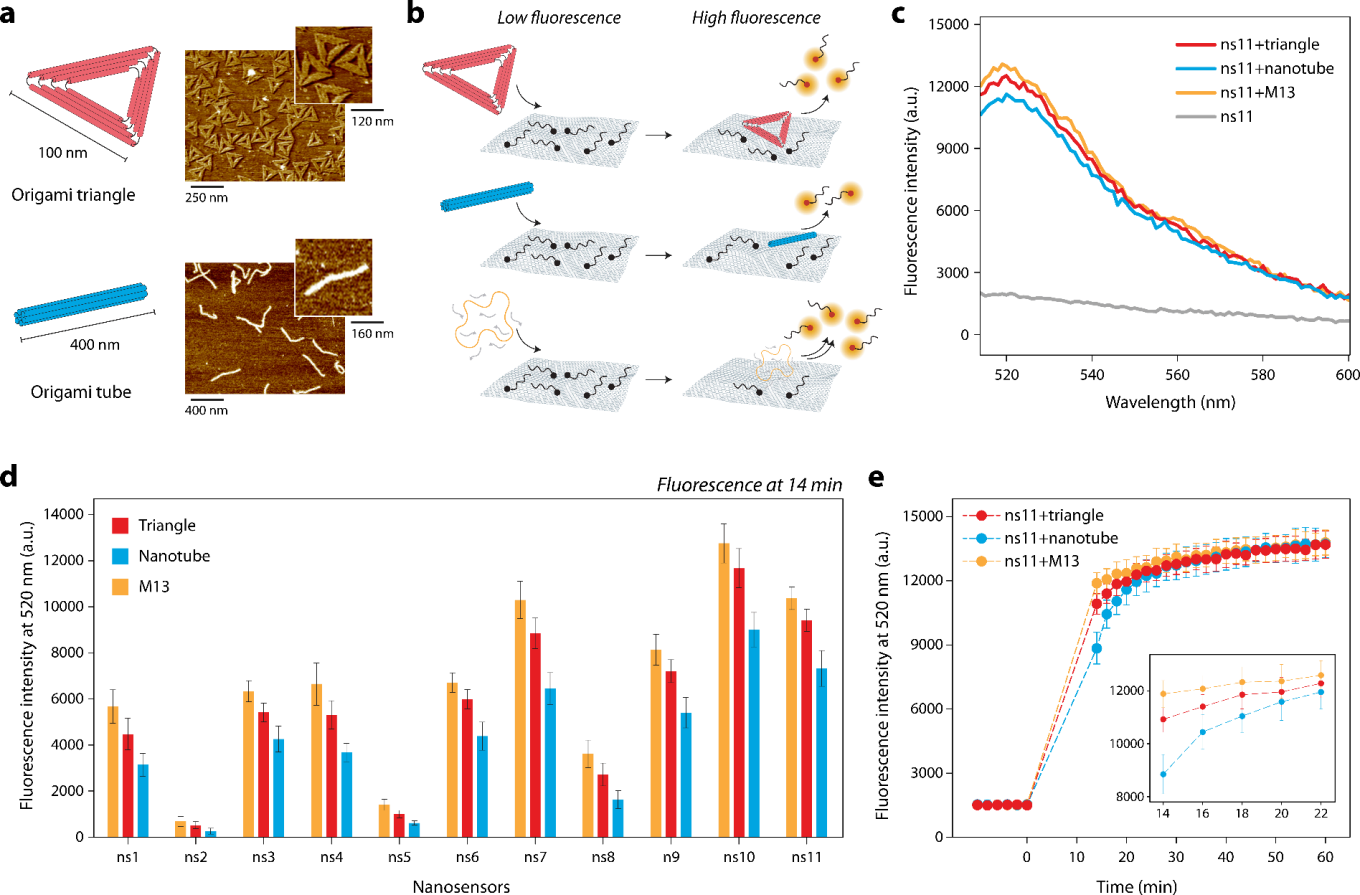
DNA nanostructure characterization and nanosensor response. (a) AFM images of the DNA origami nanostructures used for shape sorting. (b) Illustration showing the differential fluorescence recovery with each DNA origami nanostructure and M13 scaffold. (c) Fluorescence recovery from a representative nanosensor (ns11) with the addition of a DNA nanostructure or the control M13 scaffold. (d) Fingerprint fluorescence data from a nanosensor array for the DNA structures and control at the 14 min time point. (e) Kinetics of fluorescence recovery of ns11 nanosensor on the addition of DNA nanostructures. Experiments were performed in six replicates, and data are presented as mean ± SD.

Our nanostructure discrimination strategy is based on the ability of the DNA nanostructure to differentially displace the fDNA probes previously adsorbed on the nGO surface (Figure 3b). To first evaluate our strategy, we tested the response of a single nanosensor (ns11) in the presence of the DNA origami structures, showing that addition of the target DNA nanostructure resulted in fluorescence recovery (Figure 3c). This analysis was then extended to all 11 nanosensors, where fluorescence recovery over time was monitored through changes in the fluorescence spectra (Figure S3). As a control, we used a mixture of M13 scaffold and staples but with no annealing. The fluorescence intensities after the addition of the DNA origami nanostructure and M13 scaffold were plotted for each nanosensor in the array. While the random M13 nanosensor consistently showed the highest fluorescence intensity and the nanotube the lowest, their overall fingerprint patterns were similar (Figure 3d).

The resemblances in the fluorescence recovery spectra obtained with each DNA structure and similar fingerprint response trend observed across all nanosensors made us consider what additional fluorescence features could be used to improve our ability to discriminate them. All three DNA nanostructures share the same M13 genetic sequence, so relying solely on fluorescence intensity differences at a single time point carries a risk of inaccurate prediction. The collective physicochemical properties of the DNA origami including dimensions, shape, charge distribution, and diffusion behavior as well as their interactions with nGO and the fDNAs, influence the displacement kinetics of the fDNAs adsorbed on the nGO surface.^19–24^ To capture any differences in fluorescence recovery kinetics, we then recorded fluorescence response over a course of 60 min with 2 min intervals instead of collecting data points at single time point (Figure 3e for ns11 and Figure S4 for rest of the array). The data demonstrated that each sample exhibits a distinct recovery profile, which can be used to extract parameters for machine learning analysis. Analysis of the complete kinetic profiles revealed that the largest separation in fluorescence recovery rates among different DNA origami structures consistently occurred within the 14–22 min interval (Figure 4). This time window corresponds to the transition phase between the initial rapid interaction and the slower adsorption–desorption equilibrium. During this regime, displacement kinetics are most sensitive to target-dependent interactions, resulting in enhanced contrast and improved discriminatory power. Therefore, we incorporated the rate of fluorescence recovery observed between 14 and 22 min into the ML analysis, performing feature engineering within this time window to generate additional parameters for ML–assisted classification of the DNA nanostructures.

**Figure 4:**
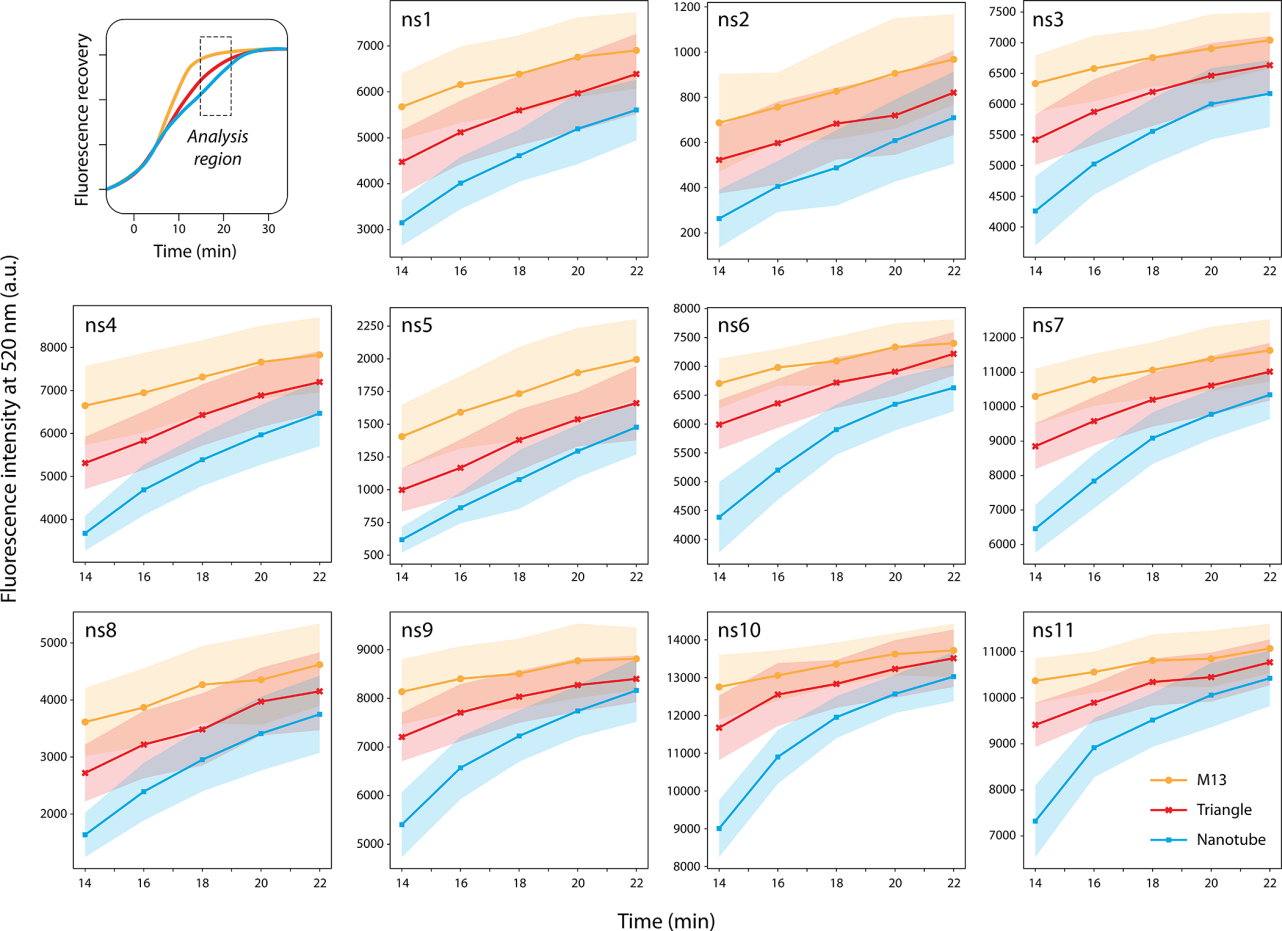
Kinetic analysis for feature engineering. Top left shows an illustration indicating the fluorescence recovery region used for feature engineering. Plots show fluorescence recovery trend observed in the 14–22 min window for DNA origami nanostructures and the M13 scaffold with each nanosensor.

To move beyond static fluorescence intensity values (Supporting Data Set 1), we found that the kinetic behavior of the sensor array response provides more informative and discriminative features for classification. To quantify these temporal dynamics, we engineered a set of 55 features based on a “Slope Model” (Supporting Data Set 2). This approach included the initial fluorescence reading from the 14 min time point for each sensor, plus the calculated slope (i.e., the change in fluorescence) between each consecutive pair of time points within the 14–22 min window (e.g., 14–16 min, 16–18 min, etc.). To distinguish among the DNA nanostructures, we employed two complementary supervised learning techniques: Random Forest (RF) classification and Partial Least Squares Discriminant Analysis (PLS-DA) using the engineered features. We first directly compared the performance of a Random Forest classifier trained on these slope-based features against an identical model trained on the raw static fluorescence data (Table 1).

**Table 1:** Cross-Validated Accuracy Comparison Demonstrating the Superior Performance of the Random Forest Model When Trained with Engineered Slope Features over Raw Data

Model/feature set	Cross-validation accuracy	Standard deviation
Raw data model	72.22	±7.86
Engineered slope model	94.44	±7.86

The model using the engineered slope features achieved a significantly higher cross-validated accuracy of 94.4%, a dramatic improvement over the 72.2% accuracy achieved by the model using raw data (GitHub repository). The RF model demonstrated excellent predictive power, achieving a weighted average F1-score of 0.94, which indicates a strong balance between precision and recall and average accuracy of 0.94 (Figure 5a). A subsequent confusion matrix, generated from cross-validated predictions, confirmed this high performance, showing only a single misclassification across all test samples (Figure 5b). This result confirms that capturing the kinetic fingerprint of the nanostructure-sensor interaction is critical for successful discrimination. Therefore, all subsequent analysis was performed using this superior, slope-based feature set. Feature engineering enables the extraction of more discriminative information than what is available from the raw fluorescence intensity values alone. Single intensity values at fixed time points differ only marginally between the two origami structures, which limits their power to separate the classes. In our case, incorporating slope-based features captures the kinetic behavior of the fluorescence recovery, specifically, how the signal changes over defined intervals (14–22 min). By taking these rates of change into account, the engineered slope features result in stronger accuracy compared to using raw fluorescence data alone. Among the 55 total features, the 15 most influential ones, ranked by their importance scores, are presented in Figure 5c.

**Figure 5:**
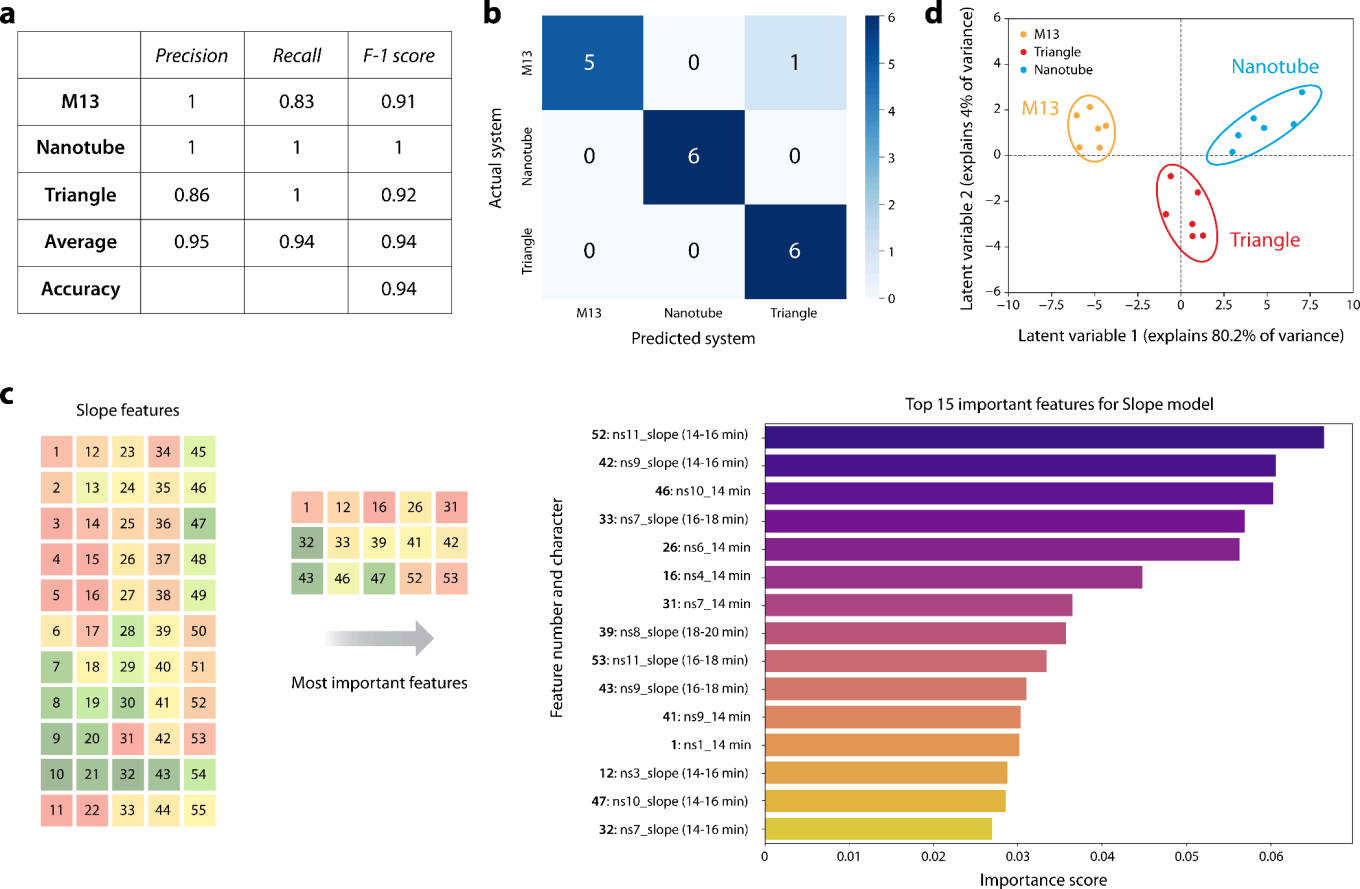
Machine learning analysis for system classification. (a) Classification model performance evaluated using F1 score, precision, and recall. (b) Confusion matrix representing the classification performance of ML-model for individual class. (c) Out of the 55 features used for slope model-based prediction, the top 15 features are ranked according to their importance score. Numbers in labels indicate the feature number. (d) PLS-DA score plot with 95% confidence ellipses.

In parallel, PLS-DA was used to reduce the dimensionality of the features and visualize the separation between the classes. The resulting score plot revealed distinct, well-separated clusters for each nanostructure, each enclosed within a 95% confidence ellipse (Figure 5d). The first two latent variables effectively captured the system’s variance, with LV1 explaining 80.2% and LV2 explaining 4% of the variance, respectively. Collectively, these engineered features form a unique “machine learning fingerprint”, enabling the accurate and reliable classification of different DNA nanostructure geometries.

In summary, we developed a fingerprinting nanosensor array combined with machine learning to effectively distinguish between two DNA origami shapes (triangle and nanotube) and to differentiate them from the random-coiled M13 genome template. The nanosensor array generated comprehensive fluorescence data sets, which were preprocessed through feature engineering to enable robust ML-based classification. The results indicate that our ML-model can classify DNA origami triangle, DNA origami nanotube and their common template (random-coiled M13 genome template) with 95% confidence ellipses in PLS-DA plot with clear separation among the systems. This model can efficiently discriminate and sort the DNA origami triangle and DNA origami nanotube based on shape with 94.4% accuracy. To maximize the information extracted from each experiment while maintaining a manageable experimental throughput, we employed only 11 nanosensors to analyze two DNA origami structures using six replicates. While this design provides sufficient data for robust analysis, further increasing the number of replicates and number of nanosensors would enable even more refined ML performance. The nanosensor array reported here inherently integrates the different properties of the DNA origami including dimensions, shape, charge distribution and processes them to generate a fluorescence recovery profile. Although it is not feasible to quantify each contributing force individually, the fluorescence recovery data provide a comprehensive aggregate signal that can be effectively analyzed using machine learning.

While our current analysis focuses on discriminating between DNA nanostructures of different geometries, the same nanosensor array and slope-based feature extraction strategy could be applied to classify other structural or nanoscale features provided that comprehensive optical data sets with discriminative features are obtained. With further optimization, our strategy could offer a broader potential for applications in monitoring reconfiguration, detecting misfolded species, or classifying unknown nanoscale assemblies in complex environments by establishing methods that are sensitive to the underlying DNA structure. Incorporating additional DNA origami structures to further increase separation between classes would likely require a broader nanosensor array and the engineered features would need to be redesigned to expand data diversity. Similarly, a generalizable ML-based shape sorting strategy would also require the ML algorithms to be re-evaluated using newly generated training and validation data sets. Therefore, any analysis involving a different set of DNA origami structures would necessitate the development of a new workflow. Our method relies on kinetic fluorescence monitoring (up to 60 min), which may be shortened or lengthened depending on the fluorescence response. The approach for this DNA origami sorting provides a practical and accessible solution using only a benchtop fluorescence reader, avoiding the need for complex instrumentation. It is also important to note that the nanosensor array does not require structural labeling, chemical modification, or individualized probe design for each origami type. Nevertheless, our work here highlights the powerful synergy between fluorescence profiling, nanosensor arrays and machine learning. It provides a platform for nanoscale feature recognition, offering new avenues for shape-sorting in DNA nanotechnology.

## Supplementary Material







## Data Availability

The detailed experimental procedure and additional data are available in the Supporting Information. The raw fluorescence intensity data set and 55 features based on a “Slope Model” are provided in Supporting Data Sets 1 and 2 (Excel format), respectively. The authors have cited additional references within the Supporting Information. The repository for the machine-learning model is available through https://github.com/AI-in-Complex-Systems-Lab/DNA_Origami_ML_Study.

## Notes

Experimental details, list of materials, oligonucleotide sequences (Tables S1–S3), and additional data (Figures S1–S4) (PDF)
Supporting Data Set 1: Raw fluorescence intensity data set (XLSX)
Supporting Data Set 2: 55 features based on a “Slope Model” (XLSX)
