# Supplementary material for: Machine Learning-Assisted DNA Origami Shape Sorting Using Fingerprinting Nanosensors and Feature Engineering
Source: Anal Chem. 2026 Jan 12;98(3):1846–53. doi: 10.1021/acs.analchem.5c06210 (PMC12856829; doi:10.1021/acs.analchem.5c06210)
Supplement: Supplementary file 3 [file ac5c06210_si_003.pdf]

Supporting Information

Machine learning (ML)-assisted DNA origami shape sorting using fingerprinting nanosensors and feature engineering

Shubhajit Singha[1], M. Mikail Demir[2], Vinod Morya[3], Ken Halvorsen[3], M. Abdullah Canbaz[2], Arun Richard Chandrasekaran[3,4,*] and Mehmet V. Yigit[1,3,*]

[1]Department of Chemistry, University at Albany, State University of New York, Albany, NY 12222.

[2]Department of Information Sciences and Technology, University at Albany, State University of New York, Albany, NY 12222.

[3]The RNA Institute, University at Albany, State University of New York, Albany, NY 12222.

[4]Department of Nanoscale Science and Engineering, University at Albany, State University of New York, Albany, NY 12222.

*Correspondence: myigit@albany.edu (MVY) and arun@albany.edu (ARC)

Table of contents

1. Experimental procedures
2. Figure S1. Fluorescence quenching of fDNA on nGO.
3. Figure S2. Formation of nanosensors (ns1–ns11) from the eleven fDNAs.
4. Figure S3. Time-dependent fluorescence spectra showing signal recovery in nanosensors.
5. Figure S4. Kinetics of fluorescence recovery on the addition of target structures.
6. Table S1. Sequences of FAM-labeled ssDNA probes (fDNAs).
7. Table S2. DNA staples used for DNA origami triangle.
8. Table S3. DNA staples used for DNA origami nanotube.

Experimental procedures

Materials. All ssDNA sequences modified with 5′ FAM were purchased from Integrated DNA Technologies (IDT, USA) and are listed in **Table S1**. M13mp18 single-stranded circular DNA was obtained from New England Biolabs (Ipswich, MA, USA; catalog no. N4040S). DNA staple sequences for the DNA triangle origami and DNA nanotube origami assemblies were also purchased from IDT (USA) and are provided in **Tables S2** and **S3**, respectively. Double-distilled water was used for buffer and oligonucleotide stock preparations. Carboxyl graphene water dispersion (used for nGO synthesis) was purchased from ACS Material (Medford, MA, USA).

Synthesis and characterization of nanographene oxide (nGO). Carboxylated graphene water dispersion was prepared according to our previous reports.[1–6] Briefly, a stock solution (5 mg/ml) of carboxyl graphene dispersion was diluted in 100 ml of double-distilled water to a final concentration of 0.5 mg/ml. The suspension was sonicated using an ultrasonic processor (Branson SLPe Digital Sonifier, Branson Ultrasonics Corp, USA) for 7 h (120 W, 20 kHz, pulse on: 2 s, pulse off: 4 s) in an ice bath to prevent overheating. The average hydrodynamic particle diameter of the obtained nGO was ~200 nm with polydispersity index (PDI) equivalent of 0.4, measured by dynamic light scattering (Zetasizer Nano-ZS, Malvern, USA).

The concentration of nGO working stocks was determined using absorbance spectra recorded on a Cary 60 UV–Vis spectrophotometer (Agilent Technologies, USA). A 100 µg/ml nGO stock solution was diluted 10-fold, yielding an OD_{230} of ~0.8. The 100 µg/ml suspension was used as the standard stock solution throughout this study. The concentration of the working stock was verified prior to each nanosensor preparation. To identify the best nGO concentration for nanosensor preparation, 20 nM of each fDNA was titrated with 0.2-1.38 µg/ml nGO concentration to observe ideal fluorescence quenching (~94%). 1.38 µg/ml of nGO was used for the subsequent fluorescence recovery studies.

Nanosensor preparation. FAM-labeled single-stranded DNA probes (5 µM) were mixed with nanographene oxide (nGO, 100 µg/ml) in buffer containing 25 mM HEPES, 100 mM NaCl, and 1 mM $MgCl_2$ (pH 7.5) for 15 min at room temperature. The interaction between fDNA and nGO was evaluated by monitoring fluorescence quenching of the FAM dye upon adsorption.[2–6] For all nanosensor formulations, the final concentrations were standardized to 20 nM DNA probe and 1.38 µg/ml nGO.

Nanosensor array preparation. The nanosensor array used in this study consisted of 11 fluorescently silent nanosensors. 500 µl of each nanosensor was prepared by incubating nanographene oxide (nGO) with fDNA (Table S1) in buffer containing 25 mM HEPES, 100 mM NaCl, and 1 mM $MgCl_2$ (pH 7.5) for 15 min. Adsorption efficiency was characterized by monitoring the decrease in fluorescence intensity of each ssDNA probe upon incubation with nGO. To improve reproducibility, nanosensor arrays were tested with each sample in six replicates, requiring a total of 6 × 11 nanosensors per sample. 50 µl of each nanosensor was added into the wells of a costar 96 well half area plate.

Synthesis and characterization of DNA origami assembly. The M13mp18 scaffold strand and staple strands corresponding to either the triangle (Table S2) or the nanotube (Table S3) were mixed at a 1:5 ratio (final DNA concentration: 5 nM) in 1× TAE buffer (40 mM Tris, 20 mM acetic acid, 1 mM EDTA) supplemented with 12.5 mM magnesium acetate. The mixture was cooled from 80 °C to room temperature over 16 h using a T100 Thermal Cycler (Bio-Rad, USA) resulting in DNA nanotube and DNA triangle structures and kept at 4 °C in the refrigerator.

Atomic force microscopy. The morphological characterization of assembled DNA origami structures was performed on Cypher-S Atomic Force Microscope (Asylum Research, Oxford Instruments) with liquid droplet probe holder and BL-AC40TS (Olympus) cantilever in tapping mode (AC mode). For imaging, freshly cleaved mica was treated with 100 mM $NiCl_2$ solution and washed with de-ionized water. 10 µl of sample was added on to the mica surface followed by washing with deposition buffer (10 mM $MgCl_2$, 25 mM KCl, 10 mM HEPES, pH 7.5) and imaged in imaging buffer (10 mM $NiCl_2$, 25 mM KCl, 10 mM HEPES, pH 7.5).

Fluorescence measurements. Fluorescence recovery was measured using a BioTek Synergy H1 microplate reader. For full-spectrum measurements, samples were excited at 485 nm, and emission spectra were recorded from 514 to 600 nm. For fluorescence kinetics studies, samples were excited at 485 nm, and emission was recorded at 520 nm at 2-min intervals over 60 minutes.

Testing the nanosensors with samples. During the initial 10 min, the fluorescence of each nanosensor in the array was monitored to assess signal stability in a costar 96-well half area plate format (50 µl nanosensor solution per well). After 10 min, each nanosensor was treated with 5 µl of 5nM DNA origami structures, and fluorescence measurements resumed immediately. Fluorescence measurements were recorded at 2-min intervals for 60 minutes.

Machine learning analysis

Data preprocessing and feature engineering. The raw fluorescence recovery data, collected at 2-minute intervals over 60 minutes, served as the input for the analysis. Upon initial inspection, the time window between 14 and 22 minutes was identified as containing the most significant variation between the experimental systems. All subsequent analyses were therefore focused on this specific time frame.

To prepare the data for modeling, it was first reshaped from a long format into a wide-format matrix using the pandas library in Python. In this matrix, each row corresponded to a unique experimental replicate, and the columns represented the fluorescence intensity of each of the 11 nanosensors at the time points of 14, 16, 18, 20, and 22 minutes.

To capture the kinetic behavior of the system, a set of slope-based features was engineered. Rather than using static fluorescence intensity values directly (Supporting Dataset 1, separate MS Excel file), we calculated the change in fluorescence between each consecutive pair of time points for every nanosensor (e.g., slope_14-16, slope_16-18, slope_18-20, and slope_20-22). The initial fluorescence value at 14 minutes was also retained as a feature. This feature engineering process resulted in a final feature matrix of 55 features (11 sensors × [1 initial value + 4 slope values]) for each experimental sample (Supporting Dataset 2, separate MS Excel file). The target variable was the system type ("DNA triangle origami," "DNA nanotube origami," etc.), which was numerically encoded for model training.

Random Forest (RF) classification. A Random Forest (RF) classifier was employed to distinguish among the three experimental systems based on the 55 engineered features. The RF model, known for its high accuracy and robustness to overfitting, was implemented using the RandomForestClassifier module from the scikit-learn library. The model was constructed with 100 decision trees (n_estimators=100), and a fixed random state (random_state=42) was used to ensure the reproducibility of the results.

Partial Least Squares Discriminant Analysis (PLS-DA). As a complementary method for visualizing the separation between the system classes in a lower-dimensional space, Partial Least Squares Discriminant Analysis (PLS-DA) was performed. This analysis was conducted using the PLSRegression module from scikit-learn, where the model was trained to predict the numerically encoded class labels from the feature matrix. The resulting scores from the first two latent variables (LVs) were plotted to visualize the clustering of the samples. 95% confidence ellipses were calculated and overlaid on the scores plot to graphically represent the separation between the groups.

Model evaluation and validation. The performance of the RF model was rigorously assessed using a stratified 3-fold cross-validation strategy, implemented via scikit-learn's StratifiedKFold. This approach ensures that the proportion of samples for each class is maintained in each fold, which is critical for evaluating performance on a balanced basis. Key performance metrics, including overall accuracy, precision, recall, and the weighted-average F1-score, were calculated from the out-of-fold predictions. A confusion matrix was also generated to visualize the classification performance for each individual class. To identify the most influential predictors, feature importances were extracted from the final RF model trained on all available data.

Software and libraries. All machine learning analyses were conducted using Python (version 3.x). The primary libraries used were pandas for data manipulation and preparation, NumPy for numerical operations, scikit-learn for all modeling and evaluation tasks, and Matplotlib, Seaborn, and Plotly for data visualization.

The repository for the machine-learning model is available through https://github.com/AI-in-Complex-Systems-Lab/DNA_Origami_ML_Study

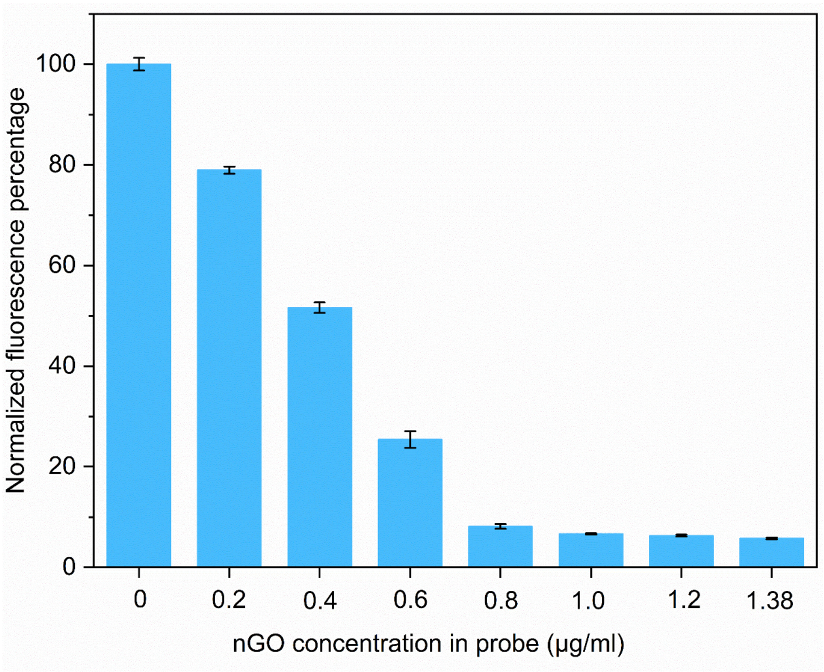

Figure S1. Fluorescence quenching of 20 nM fDNA (ns1) using varying concentrations of nGO, confirming that 1.38 µg/ml is sufficient to achieve ~94% quenching. Experiments were performed in five replicates, and data are presented as mean ± SD.

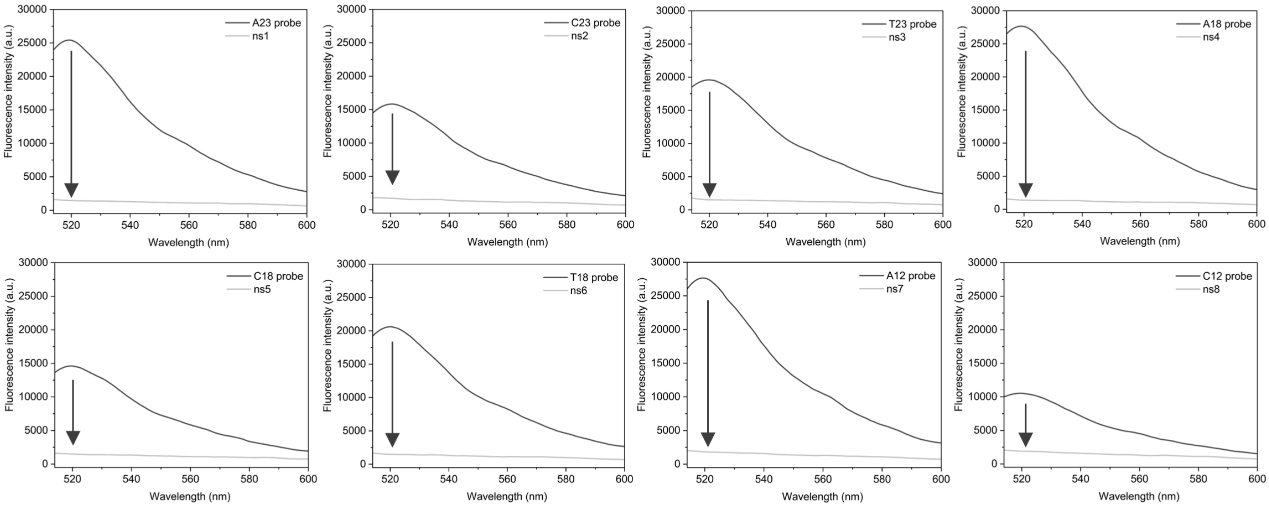

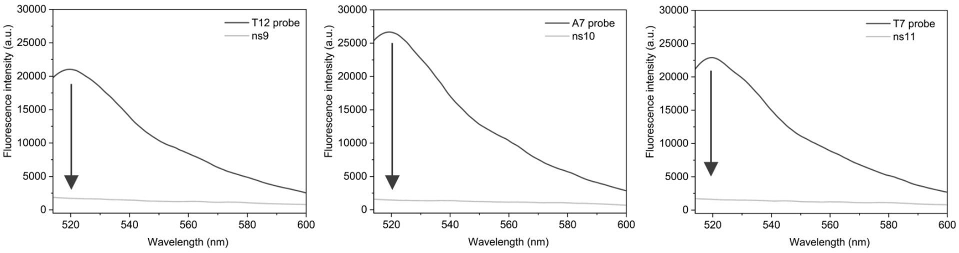

ns number	ns title
ns1	A23-nGO
ns2	C23-nGO
ns3	T23-nGO
ns4	A18-nGO
ns5	C18-nGO
ns6	T18-nGO
ns7	A12-nGO
ns8	C12-nGO
ns9	T12-nGO
ns10	A7-nGO
ns11	T7-nGO

Figure S2. Formation of nanosensors (ns1–ns11) from the eleven fDNAs. Addition of 20 nM fDNA to 1.38 μg/ml nGO reduces the fluorescence signal due to adsorption of fDNAs on the nGO.

ns number	ns title
ns1	A23-nGO
ns2	C23-nGO
ns3	T23-nGO
ns4	A18-nGO
ns5	C18-nGO
ns6	T18-nGO
ns7	A12-nGO
ns8	C12-nGO
ns9	T12-nGO
ns10	A7-nGO
ns11	T7-nGO

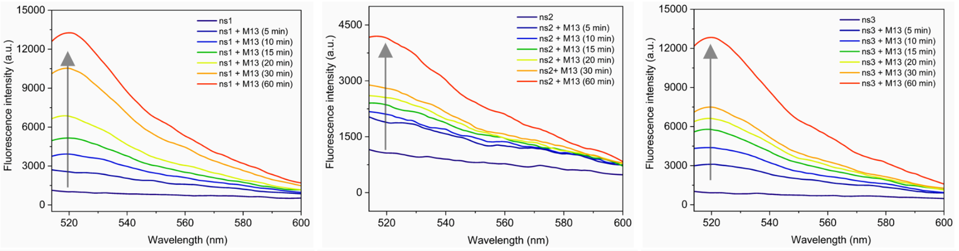

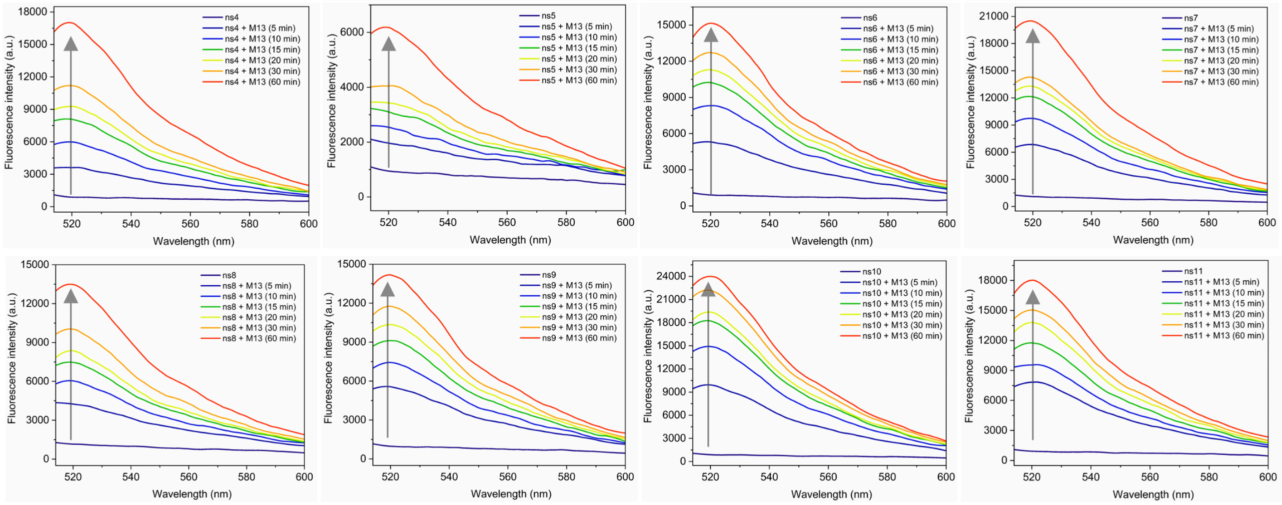

Figure S3. Representative time-dependent fluorescence spectra showing signal recovery in nanosensors (ns1-ns11) following the addition of 5 µl of 5 nM unfolded M13 to 50 µl of each nanosensor solution.

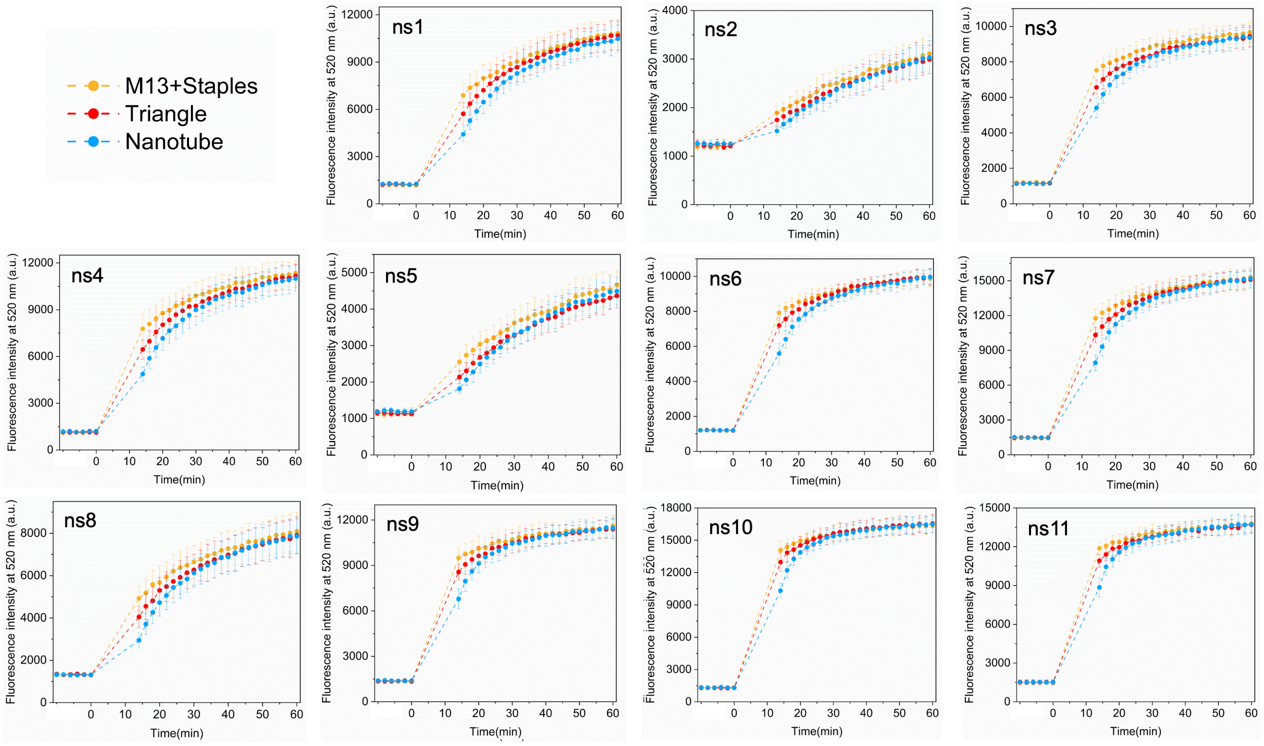

Figure S4. Kinetics of fluorescence recovery of 11 nanosensors (ns1-ns11) on the addition of target structures.

Table S1. Sequences of FAM-labeled ssDNA probes (fDNAs) written 5′-3′.

Nanosensor #	Nanosensor ID	Sequence
ns1	A23-nGO	FAM-AAAAAAAAAAAAAAAAAAAAAAA
ns2	C23-nGO	FAM-CCCCCCCCCCCCCCCCCCCCCCC
ns3	T23-nGO	FAM-TTTTTTTTTTTTTTTTTTTTTTT
ns4	A18-nGO	FAM-AAAAAAAAAAAAAAAAAA
ns5	C18-nGO	FAM-CCCCCCCCCCCCCCCCCC
ns6	T18-nGO	FAM-TTTTTTTTTTTTTTTTTT
ns7	A12-nGO	FAM-AAAAAAAAAAAA
ns8	C12-nGO	FAM-CCCCCCCCCCCC
ns9	T12-nGO	FAM-TTTTTTTTTTTT
ns10	A7-nGO	FAM-AAAAAAA
ns11	T7-nGO	FAM-TTTTTTT

Table S2. DNA staples used for DNA origami triangle.

Strand	Sequence
A_01	CGGGGTTTCCTCAAGAGAAGGATTTTGAATTA
A_02	AGCGTCATGTCTCTGAATTTACCGACTACCTT
A_03	TTCATAATCCCCTTATTAGCGTTTTTCTTACC
A_04	ATGGTTTATGTCACAATCAATAGATATTAAAC
A_05	TTTGATGATTAAGAGGCTGAGACTTGCTCAGTACCAGGCG
A_06	CCGGAACCCAGAATGGAAAGCGCAACATGGCT
A_07	AAAGACAACATTTTCGGTCATAGCCAAAATCA
A_08	GACGGGAGAATTAACTCGGAATAAGTTTATTTCCAGCGCC
A_09	GATAAGTGCCGTCGAGCTGAAACATGAAAGTATACAGGAG
A_10	TGTACTGGAAATCCTCATTAAAGCAGAGCCAC
A_11	CACCGGAAAGCGCGTTTTCATCGGAAGGGCGA
A_12	CATTCAACAAACGCAAAGACACCAGAACACCCTGAACAAA
A_13	TTTAACGGTTCGGAACCTATTATTAGGGTTGATATAAGTA
A_14	CTCAGAGCATATTCACAAACAAATTAATAAGT
A_15	GGAGGGAATTTAGCGTCAGACTGTCCGCCTCC
A_16	GTCAGAGGGTAATTGATGGCAACATATAAAAGCGATTGAG
A_17	TAGCCCGGAATAGGTGAATGCCCCCTGCCTATGGTCAGTG
A_18	CCTTGAGTCAGACGATTGGCCTTGCGCCACCC
A_19	TCAGAACCCAGAATCAAGTTTGCCGGTAAATA
A_20	TTGACGGAAATACATACATAAAGGGCGCTAATATCAGAGA
A_21	CAGAGCCAGGAGGTTGAGGCAGGTAACAGTGCCCG
A_22	ATTAAAGGCCGTAATCAGTAGCGAGCCACCCT
A_23	GATAACCCACAAGAATGTTAGCAAACGTAGAAAATTATTC
A_24	GCCGCCAGCATTGACACCACCCTC
A_25	AGAGCCGCACCATCGATAGCAGCATGAATTAT
A_26	CACCGTCACCTTATTACGCAGTATTGAGTTAAGCCCAATA
A_27	AGCCATTTAAACGTCACCAATGAACACCAGAACCA
A_28	ATAAGAGCAAGAAACATGGCATGATTAAGACTCCGACTTG
A_29	CCATTAGCAAGGCCGGGGGAATTA
A_30	GAGCCAGCGAATACCCAAAAGAACATGAAATAGCAATAGC
A_31	TATCTTACCGAAGCCCAAACGCAATAATAACGAAAATCACCAG

A_32	CAGAAGGAAACCGAGGTTTTTAAGAAAAGTAAGCAGATAGCCG
A_33	CCTTTTTTCATTTAACAATTTCATAGGATTAG
A_34	TTTAACCTATCATAGGTCTGAGAGTTCCAGTA
A_35	AGTATAAAATATGCGTTATACAAAGCCATCTT
A_36	CAAGTACCTCATTCCAAGAACGGGAAATTCAT
A_37	AGAGAATAACATAAAAACAGGGAAGCGCATTA
A_38	AAAACAAAATTAATTAAATGGAAACAGTACATTAGTGAAT
A_39	TTATCAAACCGGCTTAGGTTGGGTAAGCCTGT
A_40	TTAGTATCGCCAACGCTCAACAGTCGGCTGTC
A_41	TTTCCTTAGCACTCATCGAGAACAATAGCAGCCTTTACAG
A_42	AGAGTCAAAAATCAATATATGTGATGAAACAAACATCAAG
A_43	ACTAGAAATATATAACTATATGTACGCTGAGA
A_44	TCAATAATAGGGCTTAATTGAGAATCATAATT
A_45	AACGTCAAAAATGAAAAGCAAGCCGTTTTTATGAAACCAA
A_46	GAGCAAAAGAAGATGAGTGAATAACCTTGCTTATAGCTTA
A_47	GATTAAGAAATGCTGATGCAAATCAGAATAAA
A_48	CACCGGAATCGCCATATTTAACAAAATTTACG
A_49	AGCATGTATTTCATCGTAGGAATCAAACGATTTTTTGTTT
A_50	ACATAGCGCTGTAAATCGTCGCTATTCATTTCAATTACCT
A_51	GTTAAATACAATCGCAAGACAAAGCCTTGAAA
A_52	CCCATCCTCGCCAACATGTAATTTAATAAGGC
A_53	TCCCAATCCAAATAAGATTACCGCGCCCAATAAATAATAT
A_54	TCCCTTAGAATAACGCGAGAAAACTTTTACCGACC
A_55	GTGTGATAAGGCAGAGGCATTTTCAGTCCTGA
A_56	ACAAGAAAGCAAGCAAATCAGATAACAGCCATATTATTTA
A_57	GTTTGAAATTCAAATATATTTTAG
A_58	AATAGATAGAGCCAGTAATAAGAGATTTAATG
A_59	GCCAGTTACAAAATAATAGAAGGCTTATCCGGTTATCAAC
A_60	TTCTGACCTAAAATATAAAGTACCGACTGCAGAAC
A_61	GCGCCTGTTATTCTAAGAACGCGATTCCAGAGCCTAATTT
A_62	TCAGCTAAAAAAGGTAAAGTAATT
A_63	ACGCTAACGAGCGTCTGGCGTTTTAGCGAACCCAACATGT
A_64	ACGACAATAAATCCCGACTTGCGGGAGATCCTGAATCTTACCA
A_65	TGCTATTTTGCACCCAGCTACAATTTTGTTTTGAAGCCTTAAA
B_01	TCATATGTGTAATCGTAAAACTAGTCATTTTC
B_02	GTGAGAAAATGTGTAGGTAAAGATACAACTTT
B_03	GGCATCAAATTTGGGGCGCGAGCTAGTTAAAG
B_04	TTCGAGCTAAGACTTCAAATATCGGGAACGAG
B_05	ACAGTCAAAGAGAATCGATGAACGACCCCGGTTGATAATC
B_06	ATAGTAGTATGCAATGCCTGAGTAGGCCGGAG
B_07	AACCAGACGTTTAGCTATATTTTCTTCTACTA
B_08	GAATACCACATTCAACTTAAGAGGAAGCCCGATCAAAGCG
B_09	AGAAAAGCCCCAAAAAGAGTCTGGAGCAAACAATCACCAT
B_10	CAATATGACCCTCATATATTTTAAAGCATTAA
B_11	CATCCAATAAATGGTCAATAACCTCGGAAGCA
B_12	AACTCCAAGATTGCATCAAAAAGATAATGCAGATACATAA
B_13	CGTTCTAGTCAGGTCATTGCCTGACAGGAAGATTGTATAA
B_14	CAGGCAAGATAAAAATTTTTAGAATATTCAAC
B_15	GATTAGAGATTAGATACATTTCGCAAATCATA
B_16	CGCCAAAAGGAATTACAGTCAGAAGCAAAGCGCAGGTCAG

B_17	GCAAATATTTAAATTGAGATCTACAAAGGCTACTGATAAA
B_18	TTAATGCCTTATTTCAACGCAAGGGCAAAGAA
B_19	TTAGCAAATAGATTTAGTTTGACCAGTACCTT
B_20	TAATTGCTTTACCCTGACTATTATGAGGCATAGTAAGAGC
B_21	ATAAAGCCTTTGCGGGAGAAGCCTGGAGAGGGTAG
B_22	TAAGAGGTCAATTCTGCGAACGAGATTAAGCA
B_23	AACACTATCATAACCCATCAAAAATCAGGTCTCCTTTTGA
B_24	ATGACCCTGTAATACTTCAGAGCA
B_25	TAAAGCTATATAACAGTTGATTCCCATTTTTG
B_26	CGGATGGCACGAGAATGACCATAATCGTTTACCAGACGAC
B_27	TAATTGCTTGGAAGTTTCATTCCAAATCGGTTGTA
B_28	GATAAAAACCAAAATATTAAACAGTTCAGAAATTAGAGCT
B_29	ACTAAAGTACGGTGTCGAATATAA
B_30	TGCTGTAGATCCCCCTCAAATGCTGCGAGAGGCTTTTGCA
B_31	AAAGAAGTTTTGCCAGCATAAATATTCATTGACTCAACATGTT
B_32	AATACTGCGGAATCGTAGGGGGTAATAGTAAAATGTTTAGACT
B_33	AGGGATAGCTCAGAGCCACCACCCCATGTCAA
B_34	CAACAGTTTATGGGATTTTGCTAATCAAAAGG
B_35	GCCGCTTTGCTGAGGCTTGCAGGGGAAAAGGT
B_36	GCGCAGACTCCATGTTACTTAGCCCGTTTTAA
B_37	ACAGGTAGAAAGATTCATCAGTTGAGATTTAG
B_38	CCTCAGAACCGCCACCCAAGCCCAATAGGAACGTAAATGA
B_39	ATTTTCTGTCAGCGGAGTGAGAATACCGATAT
B_40	ATTCGGTCTGCGGGATCGTCACCCGAAATCCG
B_41	CGACCTGCGGTCAATCATAAGGGAACGGAACAACATTATT
B_42	AGACGTTACCATGTACCGTAACACCCCTCAGAACCGCCAC
B_43	CACGCATAAGAAAGGAACAACTAAGTCTTTCC
B_44	ATTGTGTCTCAGCAGCGAAAGACACCATCGCC
B_45	TTAATAAAACGAACTAACCGAACTGACCAACTCCTGATAA
B_46	AGGTTTAGTACCGCCATGAGTTTCGTCACCAGGATCTAAA
B_47	GTTTTGTCAGGAATTGCGAATAATCCGACAAT
B_48	GACAACAAGCATCGGAACGAGGGTGAGATTTG
B_49	TATCATCGTTGAAAGAGGACAGATGGAAGAAAAATCTACG
B_50	AGCGTAACTACAAACTACAACGCCTATCACCGTACTCAGG
B_51	TAGTTGCGAATTTTTTCACGTTGATCATAGTT
B_52	GTACAACGAGCAACGGCTACAGAGGATACCGA
B_53	ACCAGTCAGGACGTTGGAACGGTGTACAGACCGAAACAAA
B_54	ACAGACAGCCCAAATCTCCAAAAAAAAATTTCTTA
B_55	AACAGCTTGCTTTGAGGACTAAAGCGATTATA
B_56	CCAAGCGCAGGCGCATAGGCTGGCAGAACTGGCTCATTAT
B_57	CGAGGTGAGGCTCCAAAAGGAGCC
B_58	ACCCCCAGACTTTTTCATGAGGAACTTGCTTT
B_59	ACCTTATGCGATTTTATGACCTTCATCAAGAGCATCTTTG
B_60	CGGTTTATCAGGTTTCCATTAAACGGGAATACACT
B_61	AAAACACTTAATCTTGACAAGAACTTAATCATTGTGAATT
B_62	GGCAAAAGTAAAATACGTAATGCC
B_63	TGGTTTAATTTCAACTCGGATATTCATTACCCACGAAAGA
B_64	ACCAACCTAAAAAATCAACGTAACAAATAAATTGGGCTTGAGA
B_65	CCTGACGAGAAACACCAGAACGAGTAGGCTGCTCATTCAGTGA
Link-A1C	TTAATTAATTTTTTACCATATCAAA

Link-A2C	TTAATTTCATCTTAGACTTTACAA
Link-A3C	CTGTCCAGACGTATACCGAACGA
Link-A4C	TCAAGATTAGTGTAGCAATACT
Link-B1A	TGTAGCATTCCTTTTATAAACAGTT
Link-B2A	TTTAATTGTATTTCCACCAGAGCC
Link-B3A	ACTACGAAGGCTTAGCACCATTA
Link-B4A	ATAAGGCTTGCAACAAAGTTAC
Link-C1B	GTGGGAACAAATTTCTATTTTTGAG
Link-C2B	CGGTGCGGGCCTTCCAAAAACATT
Link-C3B	ATGAGTGAGCTTTTAAATATGCA
Link-C4B	ACTATTAAAGAGGATAGCGTCC
Loop	GCGCTTAATGCGCCGCTACAGGGC
C_01	TCGGGAGATATACAGTAACAGTACAAATAATT
C_02	CCTGATTAAAGGAGCGGAATTATCTCGGCCTC
C_03	GCAAATCACCTCAATCAATATCTGCAGGTCGA
C_04	CGACCAGTACATTGGCAGATTCACCTGATTGC
C_05	TGGCAATTTTTAACGTCAGATGAAAACAATAACGGATTCG
C_06	AAGGAATTACAAAGAAACCACCAGTCAGATGA
C_07	GGACATTCACCTCAAATATCAAACACAGTTGA
C_08	TTGACGAGCACGTATACTGAAATGGATTATTTAATAAAAG
C_09	CCTGATTGCTTTGAATTGCGTAGATTTTCAGGCATCAATA
C_10	TAATCCTGATTATCATTTTGCGGAGAGGAAGG
C_11	TTATCTAAAGCATCACCTTGCTGATGGCCAAC
C_12	AGAGATAGTTTGACGCTCAATCGTACGTGCTTTCCTCGTT
C_13	GATTATACACAGAAATAAAGAAATACCAAGTTACAAAATC
C_14	TAGGAGCATAAAAGTTTGAGTAACATTGTTTG
C_15	TGACCTGACAAATGAAAAATCTAAAATATCTT
C_16	AGAATCAGAGCGGGAGATGGAAATACCTACATAACCCTTC
C_17	GCGCAGAGGCGAATTAATTATTTGCACGTAAATTCTGAAT
C_18	AATGGAAGCGAACGTTATTAATTTCTAACAAC
C_19	TAATAGATCGCTGAGAGCCAGCAGAAGCGTAA
C_20	GAATACGTAACAGGAAAAACGCTCCTAAACAGGAGGCCGA
C_21	TCAATAGATATTAAATCCTTTGCCGGTTAGAACCT
C_22	CAATATTTGCCTGCAACAGTGCCATAGAGCCG
C_23	TTAAAGGGATTTTAGATACCGCCAGCCATTGCGGCACAGA
C_24	ACAATTCGACAACTCGTAATACAT
C_25	TTGAGGATGGTCAGTATTAACACCTTGAATGG
C_26	CTATTAGTATATCCAGAACAATATCAGGAACGGTACGCCA
C_27	CGCGAACTAAAACAGAGGTGAGGCTTAGAAGTATT
C_28	GAATCCTGAGAAGTGTATCGGCCTTGCTGGTACTTTAATG
C_29	ACCACCAGCAGAAGATGATAGCCC
C_30	TAAAACATTAGAAGAACTCAAACTTTTTATAATCAGTGAG
C_31	GCCACCGAGTAAAAGAACATCACTTGCCTGAGCGCCATTAAAA
C_32	TCTTTGATTAGTAATAGTCTGTCCATCACGCAAATTAACCGTT
C_33	CGCGTCTGATAGGAACGCCATCAACTTTTACA
C_34	AGGAAGATGGGGACGACGACAGTAATCATATT
C_35	CTCTAGAGCAAGCTTGCATGCCTGGTCAGTTG
C_36	CCTTCACCGTGAGACGGGCAACAGCAGTCACA
C_37	CGAGAAAGGAAGGGAAGCGTACTATGGTTGCT
C_38	GCTCATTTTTTAACCAGCCTTCCTGTAGCCAGGCATCTGC

C_39	CAGTTTGACGCACTCCAGCCAGCTAAACGACG
C_40	GCCAGTGCGATCCCCGGGTACCGAGTTTTTCT
C_41	TTTCACCAGCCTGGCCCTGAGAGAAAGCCGGCGAACGTGG
C_42	GTAACCGTCTTTCATCAACATTAAAATTTTTGTTAAATCA
C_43	ACGTTGTATTCCGGCACCGCTTCTGGCGCATC
C_44	CCAGGGTGGCTCGAATTCGTAATCCAGTCACG
C_45	TAGAGCTTGACGGGGAGTTGCAGCAAGCGGTCATTGGGCG
C_46	GTTAAAATTCGCATTAATGTGAGCGAGTAACACACGTTGG
C_47	TGTAGATGGGTGCCGGAAACCAGGAACGCCAG
C_48	GGTTTTCCATGGTCATAGCTGTTTGAGAGGCG
C_49	GTTTGCGTCACGCTGGTTTGCCCCAAGGGAGCCCCCGATT
C_50	GGATAGGTACCCGTCGGATTCTCCTAAACGTTAATATTTT
C_51	AGTTGGGTCAAAGCGCCATTCGCCCCGTAATG
C_52	CGCGCGGGCCTGTGTGAAATTGTTGGCGATTA
C_53	CTAAATCGGAACCCTAAGCAGGCGAAAATCCTTCGGCCAA
C_54	CGGCGGATTGAATTCAGGCTGCGCAACGGGGGATG
C_55	TGCTGCAAATCCGCTCACAATTCCCAGCTGCA
C_56	TTAATGAAGTTTGATGGTGGTTCCGAGGTGCCGTAAAGCA
C_57	TGGCGAAATGTTGGGAAGGGCGAT
C_58	TGTCGTGCACACAACATACGAGCCACGCCAGC
C_59	CAAGTTTTTTGGGGTCGAAATCGGCAAAATCCGGGAAACC
C_60	TCTTCGCTATTGGAAGCATAAAGTGTATGCCCGCT
C_61	TTCCAGTCCTTATAAATCAAAAGAGAACCATCACCCAAAT
C_62	GCGCTCACAAGCCTGGGGTGCCTA
C_63	CGATGGCCCACTACGTATAGCCCGAGATAGGGATTGCGTT
C_64	AACTCACATTATTGAGTGTTGTTCCAGAAACCGTCTATCAGGG
C_65	ACGTGGACTCCAACGTCAAAGGGCGAATTTGGAACAAGAGTCC

Table S3. DNA staples used for DNA origami nanotube.

Name	Sequence
1	TGTGTAGGTAAAGAATAGTAGTAGCATTCCTCAGAACCGCCA
2	CATCAATTCTACTATTCAAAAGGGTGAGATAGATTAGAGCCG
3	AACCAGAGCCACCAACCCTCAGAGCCGCAGCTGAAAAGGTGG
4	CCCTCAGAGCCACCCCGGAACCGCCTCCAAGTATTAGACTTT
5	TCAATAGATAATACATTTGAGGATTTAGCTCAGAGCCGCCAC
6	ACAAACAATTCGACAGCACTAACAACTAAAAGGCCGGAGACA
7	CCTTATTAGCGTTTAGCATTGACAGGAGATGGTCAATAACCT
8	AAAATATCTTTAGGAACTCGTATTAAATATCAAAATCACCGG
9	TTATTAATTTTAAAAGTTGAAAGGAATTCCGTTCTAGCTGAT
10	GTCAAATCACCATCTCATTTGGGGCGCGCACCAGAACCACCA
11	CCAGAGCCGCCGCCGCCATCTTTTCATACCTTTGCCCGAACG
12	GTTTAGCTATATTTAATATGATATTCAAGAGGAAGGTTATCT
13	CACCGTAATCAGTAAGCGCAGTCTCTGATCTGGAAGTTTCAT
14	GTTGGCAAATCAACAGTTTGAGTAACATTTCGGTCATAGCCC
15	TGATTATCAGATGAAGCAAATGAAAAATTGGAGCAAACAAGA
16	AAATTAATGCCGGAGATACATTTCGCAAGTTGAGGCAGGTCA
17	AAGCCAGAATGGAAGCGACAGAATCAAGTATCATCATATTCC
18	GACGATTGGCCTTGTTTTCATCGGCATTTATCATTTTGCGGA

19	ACAAAGAAACCACCTATCAAACCCTCAATTGAGAGATCTACA
20	TGCTGAACCTCAAAAGAAGGAGCGGAATTTTGCCTTTAGCGT
21	AAGGCTATCAGGTCGATTCCCAATTCTGATAAATCCTCATTA
22	CAGACTGTAGCGCGATATTCACAAACAACGAACGAGTAGATT
23	TAGTTTGACCATTAGAGGGTAGCTATTTTCAATATCTGGTCA
24	TCCATATAACAGTTATTGCCTGAGAGTCCTAAAGCATCACCT
25	GGGAATTAGAGCCAGGGGTCAGTGCCTTCATTTTTGCGGATG
26	CAACCGATTGAGGGAGTATTAAGAGGCTAGCTTCAAAGCGAA
27	CGCTGAGAGCCAGCTGGCAATTCATCAAACCATCGATAGCAG
28	GTACCTTTTACATCACGTGGCACAGACATCAGCTCATTTTTT
29	GAATCGATGAACGGACTAAAGTACGGTGATTTACCGTTCCAG
30	TTCTGAAACATGAAAGGGAAGGTAAATAAATATACAGTAACA
31	TAAGCGTCATACATACGTCACCAATGAATATAATCCTGATTG
32	TTTGGATTATACTTAGTATTAACACCGCGCATGTCAATCATA
33	GAGGTGAGGCGGTCCTGAATAATGGAAGAGCACCATTACCAT
34	TAATAAGTTTTAACGCAAAATCACCAGTGGTTAGAACCTACC
35	ATATCAAAATTATTGAACGAACCACCAGCCAAAAACAGGAAG
36	CCATTAAAAATACCTGCACGTAAAACAGGACTTGAGCCATTT
37	GTATAAACAGTTAATTATCACCGTCACCAAATAAAGAAATTG
38	CGTAGATTTTCAGGTGCGCGAACTGATAACGTTAATATTTTG
39	CTATTAGTCTTTAATTTAACGTCAGATGTTGACGGAAATTAT
40	TTAAAATTCGCATTAACTCCAACAGGTCTTCGGAACCTATTA
41	TCATTAAAGGTGAATGCCCCCTGCCTATAGGATTAGAGAGTA
42	ATTGTATAAGCAAATTTTGATAAGAGGTGAGTAACAGTGCCC
43	TGTACCCCGGTTGATGCTGAATATAATGCAGGAGTGTACTGG
44	GCTTAGAGCTTAATTAATCAGAAAAGCCCAGAAGATAAAACA
45	GTTTTAAATATGCATAATCGTAAAACTACTGCAACAGTGCCA
46	TAGCAAGGCCGGAAGGCTTTTGATGATACTGTAGCTCAACAT
47	CCTTTAATTGCTCCTATTTAAATTGTAAGCCCTAAAACATCG
48	CCAGACCGGAAGCAAAATTTTTGTTAAAATATTTTTGAATGG
49	GAAAGCGTAAGAATGGGAGAAACAATAAAAAAGGGCGACATT
50	TAACCCACAAGAATCCAGACGTTAGTAACCAAAAGGAATTAC
51	AACCAATAGGAACGCGCGTTTTAATTCGGAGACTCCTCAAGA
52	GTTTTGTCGTCTTTTGAGTTAAGCCCAATTATCAAAATCATA
53	GCAACTGTTGGGAAGCAACACTATCATACGTAACGATCTAAA
54	AAGACTTCAAATATCCATCAAAAATAATAACCCTTCTGACCT
55	GGTCTGAGAGACTAATCCTGAGAAGTGTGGCCTCTTCGCTAT
56	GAGGCATAGTAAGAGGGCGATCGGTGCGTTTTATAATCAGTG
57	AGGCCACCGAGTAAAGTCAATAGTGAATTAATAAGAGCAAGA
58	AAGACGCTGAGAAGAAGAGTCTGTCCATGCCATTCAGGCTGC
59	GACGACGATAAAAAGCAAAGCGCCATTCCACGCAAATTAACC
60	GTTGTAGCAATACTCCTTGAAAACATAGCGAAGCCCTTTTTA
61	TTTTCCCTTAGAATTCTTTGATTAGTAACGGCACCGCTTCTG
62	AAGTTTTGCCAGAGTCCAGCCAGCTTTCTAACATCACTTGCC
63	TGAGTAGAAGAACTGCTTCTGTAAATCGTTACCAGAAGGAAA
64	GAGTGAATAACCTTCAAACTATCGGCCTGACAGTATCGGCCT
65	TAGCGTCCAATACTTTTGAGGGGACGACTGCTGGTAATATCC
66	AGAACAATATTACCTGGAAACAGTACATCCAAAAGAACTGGC
67	ATTACCTTTTTTAAGCCAGCCATTGCAAGGGCGCATCGTAAC
68	CCCCCTCAAATGCTACGTTGGTGTAGATCAGGAAAAACGCTC

69	ATGGAAATACCTACATTAATTACATTTAGTTAGCAAACGTAG
70	ATCAAGAAAACAAAATTTTGACGCTCAACGGCGGATTGACCG
71	ATAAATCAAAAATCTCCGTGGGAACAAATCGTCTGAAATGGA
72	TTATTTACATTGGCCTGAGCAAAAGAAGTAAAAGAAACGCAA
73	TTCATTTCAATTACAGATTCACCAGTCATGTGAGCGAGTAAC
74	AAGCAAAGCGGATTTCATCAACATTAAACACGACCAGTAATA
75	AAAGGGACATTCTGAGTTACAAAATCGCACAATCAATAGAAA
76	TGCTTTGAATACCAGCCAACAGAGATAGTCGCGTCTGGCCTT
77	CCTGTAGCCAGCTTGCATCAAAAAGATTCAGTACCAGGCGGA
78	AACCCGTCGGATTCAGGTCTTTACCCTGTATAGCCCGGAATA
79	TAATGGGATAGGTCTTAAACAGTTCAGATACCGCCACCCTCA
80	CGTGCATCTGCCAGGCGGAATCGTCATACAGAGCCACCACCC
81	CAGGAAGATCGCACGGGGTAATAGTAAAGAACCCATGTACCG
82	GTGCCGGAAACCAGCCAAAATAGCGAGACTACAACGCCTGTA
83	AACAATGAAATAGCGCCCTCATAGTTAGACCCTCGTTTACCA
84	GCATTCCACAGACAAATAGCTATCTTACCGATAGCTTAGATT
85	AGAAAAGTAAGCAGGTCACCAGTACAAAGGCTTTTGCAAAAG
86	TAACACTGAGTTTCATAGCCGAACAAAGTCGCTATTAATTAA
87	CCGAGGAAACGCAAAGCAAGCCCAATAGATGTTTAGACTGGA
88	TCATTTTCAGGGATTAATAACGGAATACAAATCAATATATGT
89	ATGATTAAGACTCCAGAACCGCCACCCTAATATTCATTGAAT
90	GAACCGCCACCCTCTTATTACGCAGTATACAATTTCATTTGA
91	AAAATACATACATACTCAGGAGGTTTAGAAACGAGAATGACC
92	GGTGTATCACCGTAAAGGTGGCAACATAATGATGAAACAAAC
93	AGACACCACGGAATAGGGTTGATATAAGACTATTATAGTCAG
94	TAAGTGCCGTCGAGAAGTTTATTTTGTCGCAGAGGCGAATTA
95	ATTCATATGGTTTAAGCGGGGTTTTGCTAAGAGGAAGCCCGA
96	GAAGGATTAGGATTCCAGCGCCAAAGACCGGATTCGCCTGAT
97	AACGGTACGCCAGACCTTTTTAACCTCCTAATATCAGAGAGA
98	GAACGCGAGGCGTTGCATCGGAACGAGGAGGCTGGCTGACCT
99	TACGCCAGCTGGCGGCAGATACATAACGATGAATTTTCTGTA
100	AGCAGCGAAAGACATTAGCGAACCTCCCCCAACATGTAATTT
101	CTGCCCGCTTTCCACTTGACAAGAACCGGGATCGTCACCCTC
102	CACATTCAACTAATAAAGGGGGATGTGCGGATTTTAGACAGG
103	AGGCAGAGGCATTTCCATCACCCAAATCCGTGCCAGCTGCAT
104	TCATCAAGAGTAATGTCGGGAAACCTGTAAGTTTTTTGGGGT
105	CGAGGTGCCGTAAACATATTTAACAACGGACTTGCGGGAGGT
106	TAATTGAGAATCGCGCACTAAATCGGAATTGCGTTGCGCTCA
107	AAATCAACGTAACACTAACTCACATTAACCCTAAAGGGAGCC
108	CCCGATTTAGAGCTTATAAAGCCAACGCTATTTTGCACCCAG
109	CAAATTCTTACCAGTGACGGGGAAAGCCGTAAAGCCTGGGGT
110	CCCTGACGAGAAACCGGAAGCATAAAGTGGCGAACGTGGCGA
111	GAAAGGAAGGGAAGAAGCCTGTTTAGTAGCTAACGAGCGTCT
112	ATAATTACTAGAAAAAAGCGAAAGGAGCCGCTCACAATTCCA
113	GATGGTTTAATTTCGTGAAATTGTTATCGGGCGCTAGGGCGC
114	TGGCAAGTGTAGCGGCGTTAAATAAGAAATAAACAGCCATAT
115	GTGTGATAAATAAGGTCACGCTGCGCGTCGTAATCATGGTCA
116	CGATTTTAAGAACTACCGAGCTCGAATTAACCACCACACCCG
117	CCGCGCTTAATGCGCTAAATTTAATGGTTTTTTTGTTTAACG
118	TTTCATCTTCTGACCCGCTACAGGGCGCGCAGGTCGACTCTA

119	AAGAAAAATCTACGAAGCTTGCATGCCTGTACTATGGTTGCT
120	TTGACGAGCACGTAAAAACTTTTTCAAAAGAGAATAACATAA
121	ACAAAGAACGCGAGTAACGTGCTTTCCTACGACGTTGTAAAA
122	TATTACAGGTAGAAGGGTTTTCCCAGTCCGTTAGAATCAGAG
123	CGGGAGCTAAACAGTAAATGCTGATGCATTAACTGAACACCC
124	TATATAACTATATGGAGGCCGATTAAAGTGCAAGGCGATTAA
125	GTTGGGTAACGCCAAGATTCATCAGTTGTTTCAGCGGAGTGA
126	CGACGGCCAGTGCCTTAATAAAACGAACCGAATAATAATTTT
127	GAGGATCCCCGGGTGGCTCATTATACCACTCCAAAAGGAGCC
128	TAGCTGTTTCCTGTAACTTTAATCATTGTTCGAGGTGAATTT
129	CACAACATACGAGCACCAGAACGAGTAGCCGACAATGACAAC
130	GCCTAATGAGTGAGAAGCTGCTCATTCATCGGTCGCTGAGGC
131	TTTGAAGCCTTAAAAGGCCGCTTTTGCGGATATTCATTACCC
132	TTGCAGGGAGTTAATCAAGATTAGTTGCTCAACAGTAGGGCT
133	CTACAATTTTATCCCATAACCGATATATGTGAATAAGGCTTG
134	AACCATCGCCCACGTGAATCTTACCAACTCATATGCGTTATA
135	TTCCAGAGCCTAATTACCGATAGTTGCGTAAATTGGGCTTGA
136	CTTAAACAGCTTGATTGCCAGTTACAAATAAACACCGGAATC
137	TATTTATCCCAATCTTTATCAGCTTGCTTGAATTACCTTATG
138	TTTAATTGTATCGGCAAATAAGAAACGATTGAAATACCGACC
139	TCAAAAATGAAAATCTCCAAAAAAAAGGGTCAGGACGTTGGG
140	TTCACGTTGAAAATAGCAGCCTTTACAGTATATTTTAGTTAA
141	AAACAGGGAAGCGCAACTAAAGGAATTGTAACGGAACAACAT
142	GAATAGAAAGGAACATTAGACGGGAGAAAATCCAATCGCAAG
143	TGAACAAAGTCAGAACAACTTTCAACAGAGATTTAGGAATAC
144	TGGGATTTTGCTAAGGGTAATTGAGCGCGGCTTAGGTTGGGT
145	GCCCACTACGTGAATCGAGCCAGTAATAATCCGGTATTCTAA
146	TAATGAATCGGCCACAGACCAGGCGCATGTAGCAACGGCTAC
147	AGATGAACGGTGTAACGCGCGGGGAGAGCTATCAGGGCGATG
148	TTTACGAGCATGTATTTGATGGTGGTTCAGGCGAAAATCCTG
149	CGGAGATTTGTATCTGGTTTGCCCCAGCCGAAATCGGCAAAA
150	TCCCTTATAAATCAGAACAAGAAAAATACTTATCATTCCAAG
151	AATAGATAAGTCCTAAAGAATAGCCCGATGAGAGAGTTGCAG
152	CGACCTGCTCCATGTCACCGCCTGGCCCGATAGGGTTGAGTG
153	TTGTTCCAGTTTGGCTAATGCAGAACGCATCGAGAACAAGCA
154	AACAACATGTTCAGAACAAGAGTCCACTAGTGAGACGGGCAA
155	TCAATCATAAGGGATTTTTCTTTTCACCATTAAAGAACGTGG
156	ACTCCAACGTCAAAAAGTAATTCTGTCCATTACCGCGCCCAA
157	ACCGACAAAAGGTAGGGCGAAAAACCGTGCGGTTTGCGTATT
158	GGGCGCCAGGGTGGACCGAACTGACCAAATGAGGAAGTTTCC
159	CAGCTGATTGCCCTTTACTTAGCCGGAATACGAAGGCACCAA
160	CAAGCGGTCCACGCATCGCCTGATAAATCTAAAACACTCATC
161	GAAACCAATCAATAATTATACCAAGCGCGAAACAAAGTACAA
162	TTTGACCCCCAGCGATCGGCTGTCTTTCATATCCCATCCTAA
163	AACGGGTATTAAACGGCAAAAGAATACATGTGTCGAAATCCG
164	CCTAAAACGAAAGACAAGTACCGCACTCGCCTGTTTATCAAC
165	AGCCGTTTTTATTTATACGTAATGCCACCGAGGCGCAGACGG
166	ATTAAACGGGTAAATCATCGTAGGAATCAGACGACGACAATA
167	TAGCAAGCAAATCACTAAAGACTTTTTCCTTTGAAAGAGGAC
168	AGAGGCTTTGAGGAGATATAGAAGGCTTAGAGAATATAAAGT

References

1. Balcioglu, M., Rana, M., Robertson, N. & Yigit, M. V. DNA-Length-Dependent Quenching of Fluorescently Labeled Iron Oxide Nanoparticles with Gold, Graphene Oxide and MoS2 Nanostructures. *ACS Appl. Mater. Interfaces* **6**, 12100–12110 (2014).
2. Hizir, M. S. *et al.* Universal sensor array for highly selective system identification using two-dimensional nanoparticles. *Chem. Sci.* **8**, 5735–5745 (2017).
3. Hizir, M. S., Nandu, N. & Yigit, M. V. Homologous miRNA Analyses Using a Combinatorial Nanosensor Array with Two-Dimensional Nanoparticles. *Anal. Chem.* **90**, 6300–6306 (2018).
4. Nandu, N., Hizir, M. S. & Yigit, M. V. Systematic Investigation of Two-Dimensional DNA Nanoassemblies for Construction of a Nonspecific Sensor Array. *Langmuir* **34**, 14983–14992 (2018).
5. Smith, C. W., Hizir, M. S., Nandu, N. & Yigit, M. V. Algorithmically Guided Optical Nanosensor Selector (AGONS): Guiding Data Acquisition, Processing, and Discrimination for Biological Sampling. *Anal. Chem.* **94**, 1195–1202 (2022).
6. Smith, C. W., Kachwala, M. J., Cole, R. L. & Yigit, M. V. Machine Learning Enabled Nanosensor Array for Monitoring Citrus Juice Adulteration. *ACS Food Sci. Technol.* **2**, 1217–1223 (2022)
